# Supplementary material for: Dysbindin links presynaptic proteasome function to homeostatic recruitment of low release probability vesicles
Source: Nat Commun. 2018 Jan 18;9:267. doi: 10.1038/s41467-017-02494-0 (PMC5773495; doi:10.1038/s41467-017-02494-0)
Supplement: Supplementary file 1 — Supplementary Information [file 41467_2017_2494_MOESM1_ESM.pdf]

SUPPLEMENTARY FIGURES

Wentzel *et al.*

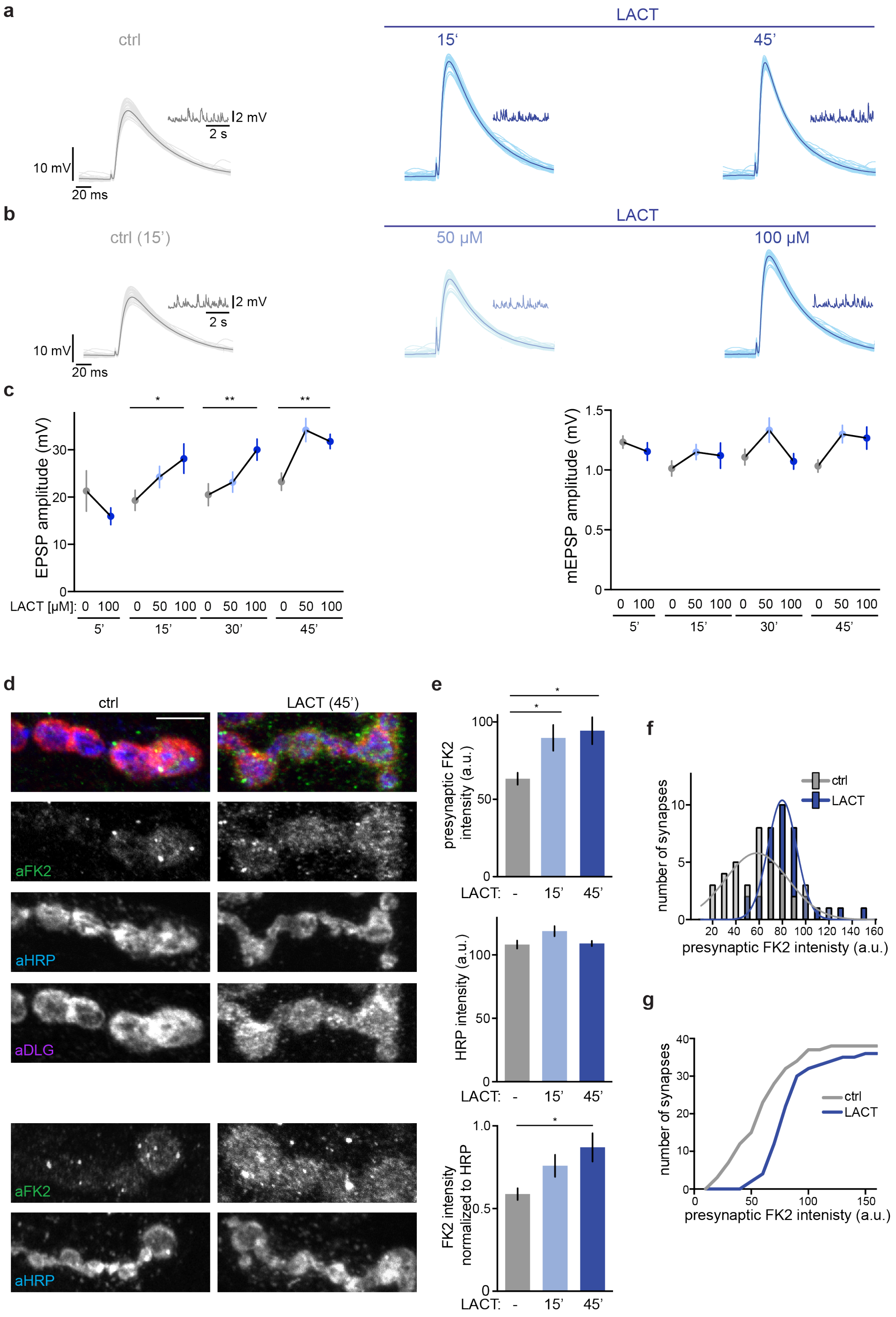

Supplementary Fig. 1: Proteasome inhibition rapidly increases neurotransmitter release

(**a**) Representative EPSP and mEPSP traces showing the increase in EPSP amplitude after application of 100 μM lactacystin for different durations at 0.3 mM $[Ca^{2+}]_e$. mEPSP amplitude or frequency is not changed. Controls were incubated with saline for 45 min. (**b**) Representative traces showing that the increase in EPSP amplitude scales with an increase in lactacystin concentration. Larvae were incubated with lactacystin or saline (control) for 15 min. (**c**) Quantification of EPSP amplitude and mEPSP amplitude after incubation of larvae with lactacystin for the indicated durations and lactacystin concentrations. EPSP amplitude increases with lactacystin concentration. mEPSP amplitude does not change significantly after lactacystin application. Mean ± s.e.m.; n ≥ 10 cells; $^*p < 0.05$; $^{**}p < 0.001$; Student's t-test. (**d**) Representative immunostainings of larval neuromuscular junctions after lactacystin (100 μM) or saline incubation for 45 min. Stainings with an antibody against mono- and polyubiquitinated proteins (aFK2), neuronal membranes (aHRP, anti-horseradish peroxidase), postsynaptic reticulum (aDLG, anti-discs large), and the overlay are shown; scale bar = 5 μm. The four images below show an additional example of an NMJ stained with FK2 and anti-HRP after saline or lactacystin incubation. (**e**) Top: Quantification of FK2 fluorescence intensity in the HRP mask (presynapse, see Methods) after incubation with lactacystin for the indicated durations. Mean ± s.e.m.; n = 8 cells; $^*p < 0.05$; ANOVA and Tukey's multiple comparison tests. Middle and bottom: Quantification of HRP-fluorescence intensity and FK2 intensity normalized to HRP-fluorescence intensity. Mean ± s.e.m.; n = 8 cells; $^*p < 0.05$; ANOVA and Tukey's multiple comparison tests. (**f**) FK2-fluorescence intensity distribution of control synapses (saline incubation; pooled data for all incubation times; gray) and lactacystin-treated synapses (pooled data for all incubation times, 50 or 100 μM; blue). Distributions were fitted with a Gaussian. Note the right shift and the narrowing of the distribution upon lactacystin treatment, indicating increased levels of ubiquitin-tagged proteins. (**g**) Cumulative distribution of the number of synapses over presynaptic FK2 fluorescence intensity for control synapses and after incubation with lactacystin. Mean ± s.e.m.; n = 38 synapses (ctrl), 36 synapses (LACT).

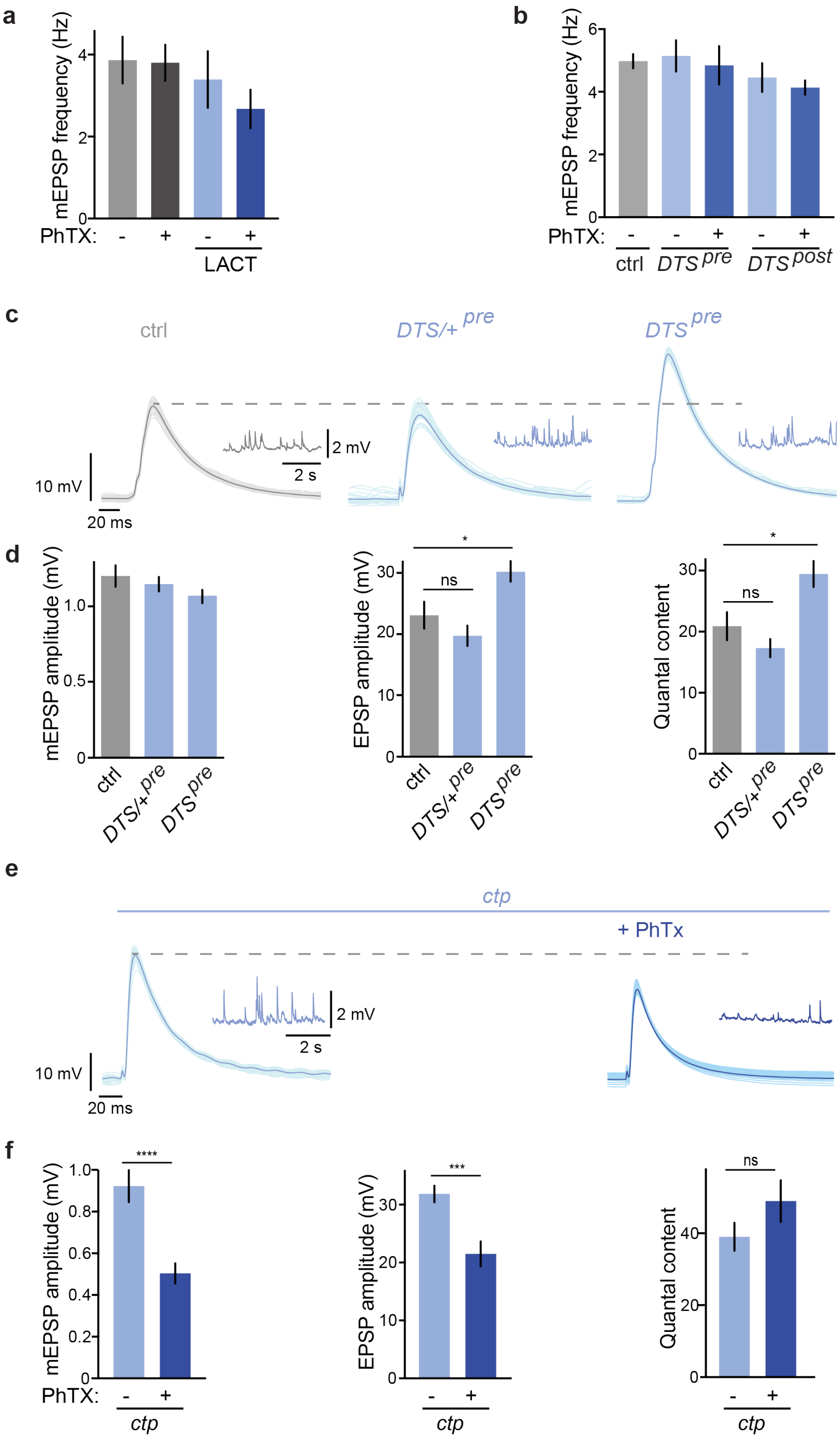
a
mEPSP frequency (Hz)
PhTX: - + - +
LACT
b
mEPSP frequency (Hz)
PhTX: - - + - +
ctrl
DTS pre
DTS post
c
ctrl
DTS/+ pre
DTS pre
2 mV
2 s
10 mV
20 ms
d
mEPSP amplitude (mV)
EPSP amplitude (mV)
Quantal content
ns
*
ctrl
DTS/+ pre
DTS pre
e
ctp
+ PhTx
2 mV
2 s
10 mV
20 ms
f
mEPSP amplitude (mV)
EPSP amplitude (mV)
Quantal content
****
***
ns
PhTX: - +
ctp

Supplementary Fig. 2: Unchanged mEPSP frequency after proteasome perturbation, effects of *DTS*-copy number on release and loss of *cut-up* disrupts PHP

(**a**) Quantification of mEPSP frequency for the experiments shown in Figure 1a and b. There is no significant change in mEPSP frequency. Mean ± s.e.m.; n ≥ 6 cells. (**b**) Quantification of mEPSP frequency for the experiment shown in Figure 1c. There is no significant change in mEPSP frequency. Mean ± s.e.m.; n ≥ 10 cells. (**c**) Representative mEPSP and EPSP traces of ctrl synapses (neuronal driver line $elav^{c155}$ only) and synapses in which one copy ($DTS/+^{pre}$) or two copies (DTS^{pre}) of the mutant proteasome subunit *DTS5* were overexpressed pan-neuronally (0.3 mM $[Ca^{2+}]_e$). (**d**) Quantification of mEPSP amplitude, EPSP amplitude and quantal content for the experiment in (c). Whereas presynaptic overexpression of one *DTS5* copy does not induce significant changes in any of these parameters, overexpression of two *DTS5* copies results in a significant increase in EPSP amplitude and quantal content. These data suggest dose-dependent effects of *DTS5* overexpression on neurotransmitter release. Mean ± s.e.m.; n ≥ 18 cells. *p < 0.05; ns: not significant; ANOVA and Dunn's multiple comparison tests. (**e**) Representative EPSP and mEPSP traces of *cut-up* (*ctp*) mutant synapses after application of saline or 20 μM PhTX for 10 min. *ctp* encodes for the *Drosophila* homologue of the dynein light chain subunit LC8, and loss of *ctp* was shown to disrupt proteasome trafficking at the *Drosophila* NMJ[1]. (**f**) Quantification of the experiment shown in (e). *ctp* mutants have a defect in presynaptic homeostatic plasticity (PHP). Mean ± s.e.m.; n ≥ 17 cells; ***p < 0.0001; ****p < 0.00001; ns: not significant; Student's t-test.

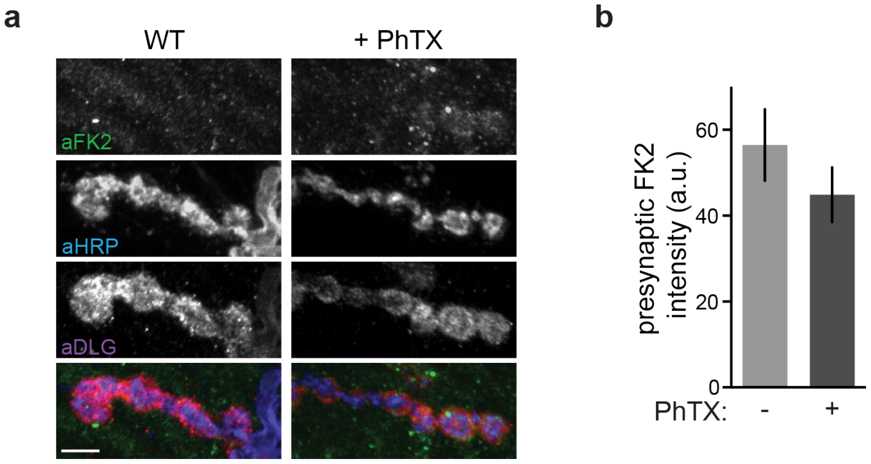

Supplementary Fig. 3: PhTX application does not enhance ubiquitinated protein levels

(**a**) Representative wild-type NMJs stained for mono- and polyubiquitinated proteins (aFK2), neuronal membrane (aHRP) and postsynaptic reticulum (aDLG), after saline or PhTX treatment (20 μM for 10 min); scale bar = 5 μm. (**b**) Quantification of FK2-fluorescence intensity in the HRP-mask (presynapse). We did not detect significant changes in FK2-fluorescence

intensity between PhTX-treated and control synapses, implying that mono- and/or polyubiquitinated protein levels are not changed upon PhTX application for 10 min. Mean ± s.e.m.; n = 16 cells.

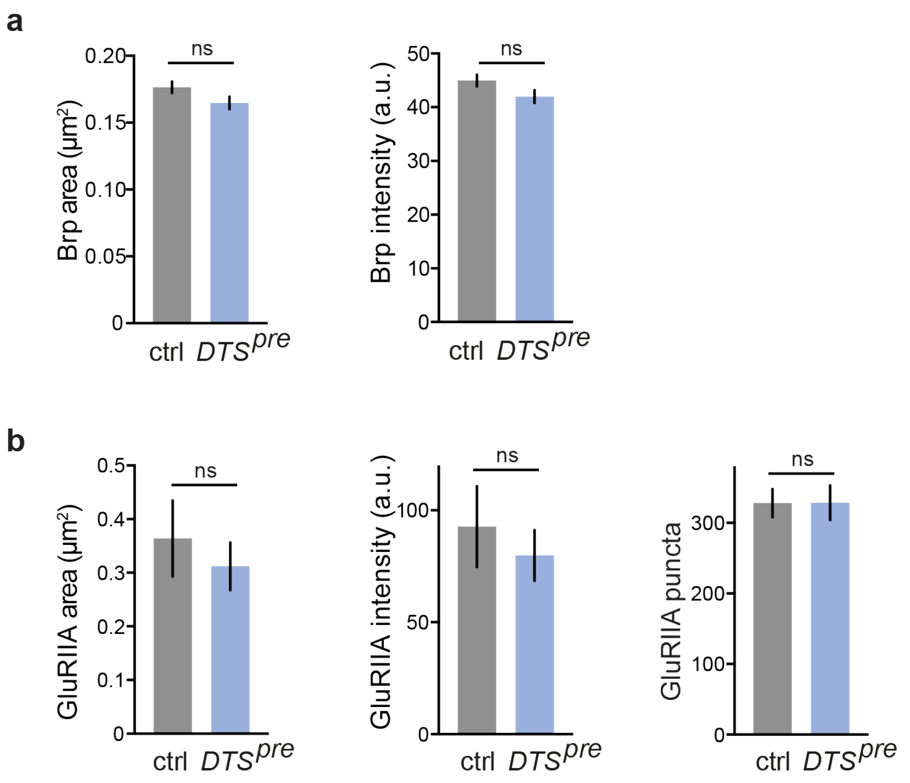

Supplementary Fig. 4: Presynaptic overexpression of *DTS5* does not induce changes in synapse morphology

(**a**) Quantification of Brp (Bruchpilot, anti-nc82) puncta area and intensity for the experiment shown in Figure 3. Presynaptic overexpression of the mutant proteasome subunit *DTS5* (*elav^{c155}; DTS5, 'DTS^{pre}'*) does not induce apparent changes in Brp area or intensity with respect to controls (*elav^{c155}*). Mean ± s.e.m.; n = 24 NMJs. (**b**) Quantification of GluRIIA area, intensity and puncta number for the experiment shown in Figure 3. There are no apparent changes after *DTS5* overexpression. Mean ± s.e.m.; n = 24 NMJs.

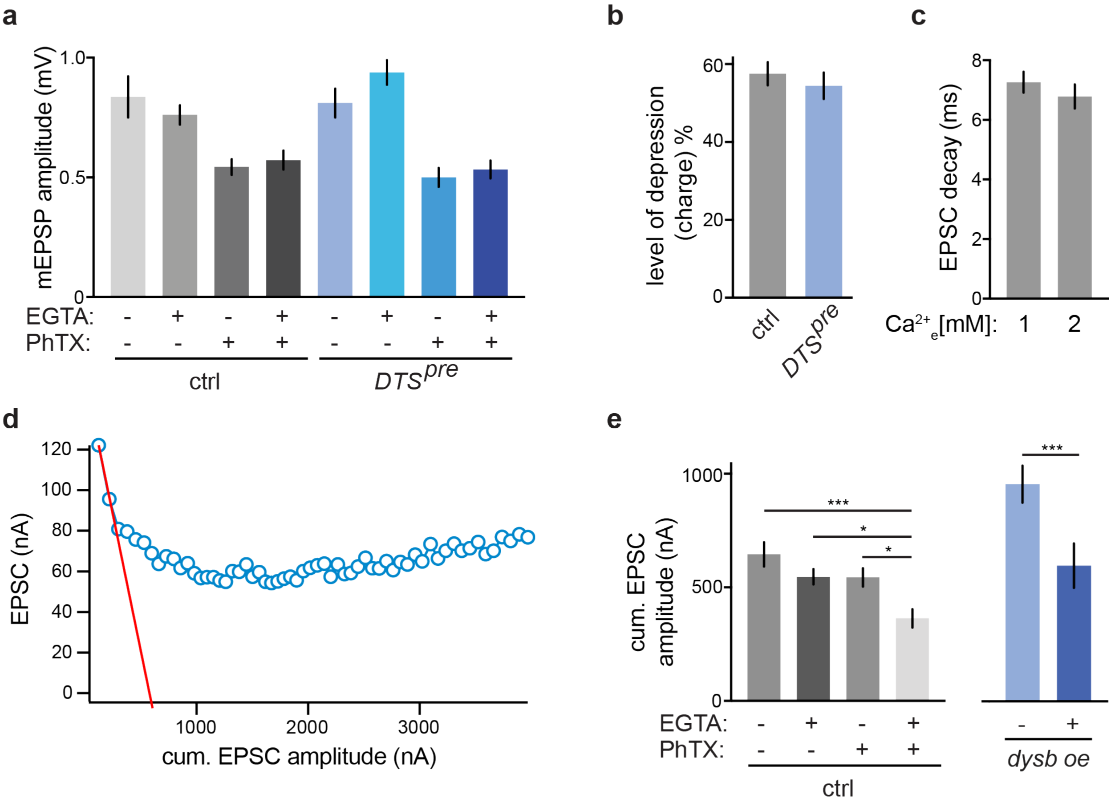

Supplementary Fig. 5: EGTA-AM application decreases cumulative EPSC amplitude after PhTX treatment and *dysbindin* overexpression
(**a**) Quantification of mEPSP amplitude for the experiment shown in Figure 5. EGTA-AM application has no effect on mEPSP amplitude compared to baseline, whereas PhTX application reduces mEPSP amplitude, as shown previously[2]. (**b**) Short-term depression of EPSC charge during a 60-Hz train (data from Figure 6 e and f; percentage of average of last three EPSC amplitudes/ first EPSC amplitude) is not different between control and DTS^{pre}, indicating no apparent changes in p_r. (**c**) Quantification of EPSC decay time constants in control larvae at 1 mM and 2 mM $[Ca^{2+}]_e$. EPSC decay time constant does not change with an increase in $[Ca^{2+}]_e$, implying that the slower EPSC decay seen in DTS^{pre} is unlikely due to changes in p_r alone. (**d**) Cumulative EPSC amplitude was calculated with the Elmqvist and Quastel (EQ) method[3,4]. Here, a linear fit to the first two points of a plot of EPSC amplitude vs. cumulative EPSC amplitude was applied. EGTA-AM application resulted in a non-uniform decrease in EPSC amplitude during the 60-Hz train, so that the 'train method'[4], which was used to estimate RRP size in Figure 6e and f, could not be applied. (**e**) Quantification of cumulative EPSC amplitudes for the experiments shown in Figure 5 and 8 of the main text (gray and blue bars, respectively). At wild-type synapses, cumulative EPSC amplitudes are significantly decreased after concomitant EGTA-AM and PhTX treatment compared to all other groups (gray bars), indicating a block of homeostatic potentiation of RRP size. *dysbindin* overexpression results in an increase in cumulative EPSC amplitude and EGTA-sensitivity (blue data) compared to controls. Mean ± s.e.m.; left: n ≥ 7 cells; $^*p < 0.05$,

***p < 0.0001; ANOVA and Tukey's multiple comparison tests; right: n ≥ 9 cells; ***p < 0.0001; Mann-Whitney test.

SUPPLEMENTARY TABLE

Genotype	$[Ca^{2+}]_e$	lactacystin (µM)	mean EPSP ampl (mV) (s.e.m.)	mean mEPSP ampl (mV) (s.e.m.)	mean QC (s.e.m.)	n
w^{1118}	0.3	-	21.16 (2.118)	0.77 (0.041)	27.69 (2.642)	10
		100 µM; 15 min	27.36 (2.092)	0.84 (0.042)	33.13 (2.162)	18
RIM^{103}	0.3	-	7.319 (0.985)	0.793 (0.058)	9.306 (1.261)	17
		100 µM; 15 min	6.664 (1.017)	0.866 (0.094)	8.442 (1.682)	13
rbp^{STOP1}/ *rbp*$^{S2.01}$	1	-	10.15 (1.326)	0.793 (0.046)	13.65 (2.176)	15
		100 µM; 15 min	14.49 (1.785)	0.756 (0.038)	19.62 (2.556)	18
dysbindin	0.3	-	22.21 (3.937)	1.37 (0.119)	15.77 (2.711)	10
		100 µM; 15 min	20.71 (2.591)	1.39 (0.069)	16.06 (2.354)	15
rab3-GAP	0.3	-	14.16 (2.452)	1.084 (0.11)	13.73 (2.373)	9
		100 µM; 15 min	22.65 (3.911)	1.18 (0.049)	19.01 (3.052)	8
cac^{s}	0.5	-	12.3 (1.328)	1.298 (0.087)	10.91 (1.452)	26
		100 µM; 15 min	15.98 (1.236)	1.251 (0.172)	15.08 (1.926)	13
snapin^{RNAi}	0.3	-	26.22 (4.113)	0.825 (0.047)	31.69 (4.389)	8
		100 µM; 15 min	34.11 (2.689)	0.897 (0.068)	40.16 (4.960)	9
ppk16^{166}	0.25	-	13.25 (1.497)	0.873 (0.05)	15.64 (1.755)	14
		100 µM; 15 min	24.2 (2.513)	0.873 (0.046)	28.02 (2.869)	12
exn^{EY10953}	0.25	-	17.4 (2.215)	1.236 (0.067)	14.45 (2.008)	17
		100 µM; 15 min	23.58 (1.437)	1.196 (0.054)	20 (1.207)	13
fife^{EX1027}	0.3	-	10.35 (1.67)	0.84 (0.05)	12.27 (1.84)	10
		100 µM; 15 min	17.39 (1.76)	0.82 (0.04)	21.73 (2.39)	10

Supplementary Table 1: Average electrophysiology data of PHP mutants after lactacystin application.
Average EPSP amplitudes, mEPSP amplitudes and quantal content values (QC) in the absence and presence of lactacystin (100 µM; 15 min) for the normalized data shown in figures 7a and 7b. The extracellular Ca^{2+} concentration ($[Ca^{2+}]_e$) was adjusted to obtain similar EPSP amplitudes in the absence of lactacystin treatment, i.e. to normalize for defects in baseline synaptic transmission of some of the mutants (references to all mutants are given in the main text). *n* refers to the number of NMJs for each experimental condition (Mean ± s.e.m.).

SUPPLEMENTARY REFERENCES

1. Kreko-Pierce, T. & Eaton, B. A. The Drosophila LC8 homologue Cut-up specifies the axonal transport of proteasomes. J. Cell Sci. 130, 3388–3398 (2017).

2. Frank, C. A., Kennedy, M. J., Goold, C. P., Marek, K. W. & Davis, G. W. Mechanisms underlying the rapid induction and sustained expression of synaptic homeostasis. Neuron 52, 663–677 (2006).

3. Elmqvist, D. & Quastel, D. M. A quantitative study of end-plate potentials in isolated human muscle. J. Physiol. 178, 505–529 (1965).

4. Thanawala, M. S. & Regehr, W. G. Determining synaptic parameters using high-frequency activation. J. Neurosci. Methods 264, 136–152 (2016).
